# Humans choose representatives who enforce cooperation in social dilemmas through extortion

**DOI:** 10.1038/ncomms10915

**Published:** 2016-03-07

**Authors:** Manfred Milinski, Christian Hilbe, Dirk Semmann, Ralf Sommerfeld, Jochem Marotzke

**Affiliations:** 1 Department of Evolutionary Ecology, Max-Planck-Institute for Evolutionary Biology, August-Thienemann-Strasse 2, 24306 Plön, Germany; 2 Department of Organismic and Evolutionary Biology, Department of Mathematics, Program for Evolutionary Dynamics, Harvard University, One Brattle Square, Cambridge, Massachusetts 02138, USA; 3 Institute of Science and Technology Austria, Am Campus 1, Klosterneuburg 3400, Austria; 4 Max Planck Institute for Meteorology, Department “The Ocean in the Earth System”, 20146 Hamburg, Germany

## Abstract

Social dilemmas force players to balance between personal and collective gain. In many dilemmas, such as elected governments negotiating climate-change mitigation measures, the decisions are made not by individual players but by their representatives. However, the behaviour of representatives in social dilemmas has not been investigated experimentally. Here inspired by the negotiations for greenhouse-gas emissions reductions, we experimentally study a collective-risk social dilemma that involves representatives deciding on behalf of their fellow group members. Representatives can be re-elected or voted out after each consecutive collective-risk game. Selfish players are preferentially elected and are hence found most frequently in the ‘representatives' treatment. Across all treatments, we identify the selfish players as extortioners. As predicted by our mathematical model, their steadfast strategies enforce cooperation from fair players who finally compensate almost completely the deficit caused by the extortionate co-players. Everybody gains, but the extortionate representatives and their groups gain the most.

## Abstract (web summary)

Social dilemmas force players to balance between personal and collective gain. Here, inspired by the negotiations for greenhouse-gas emission limitations, the authors experimentally studied a representative-based collective-risk scenario, reporting the emergence of extortionate zero-determinant (ZD) strategies.

## Graphical abstract

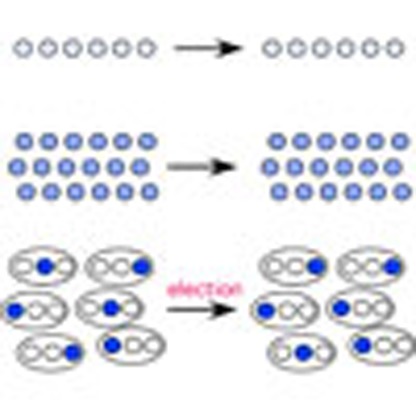

Although humans are regarded as champions of cooperation^1^,^2^, there are social dilemmas that so far have defied solution—we have not yet collaborated successfully to stop the increase of global greenhouse-gas emissions^3^,^4^, Europe continues to overexploit its marine fish stock^5^ and the European Union has so far failed to reach an equitable solution to accommodating the large number of refugees arriving from Africa and the Middle East^6^. In these and other dilemmas, essential decisions are made not by individual social actors but by representatives such as officials from elected governments. Representatives have been shown to display a more competitive mindset than ‘ordinary' group members^7^. However, the behaviour of representatives in a social dilemma has, to our knowledge, not been investigated experimentally. To fill this gap is the aim of our paper.

While we believe that our results apply to the role of representatives in social dilemmas more broadly, we have drawn our main inspiration and the concrete setting of our experiments from the challenge to prevent ‘dangerous anthropogenic interference with the climate system'^8^. This challenge is now usually interpreted as limiting global warming to below 2 °C compared with the pre-industrial period. To prevent temperature from exceeding this limit, greenhouse-gas emissions should be reduced from about 2020 onwards; by 2050, emissions should fall to a level of ≤50% of the year 2000 emissions^4^,^9^,^10^,^11^,^12^. However, as representatives attend climate summits to negotiate their country's share in reducing greenhouse-gas emissions, they are eagerly watched by their voters who might not re-elect their representatives when others negotiate a lower share^13^. Though everybody profits only if dangerous climate change is averted, none of the many climate summits has achieved sustained emissions reductions, the relative success of the Paris negotiations at COP21 notwithstanding.

The global emissions-reduction problem has been simulated experimentally in the ‘collective-risk social dilemma' game^14^,^15^,^16^,^17^,^18^. A number of volunteers can invest anonymously from their individual endowments into a climate account in each of 10 consecutive rounds. If the group collectively reaches a specified target sum, everybody receives in cash what she has not invested from her endowment. However, if the group fails to reach the target, individuals risk losing all their remaining endowment with a high probability, mimicking the drastic economic losses that result from dangerous climate change. The social dilemma arises because all players benefit only if the collective target is reached, but individual payoff is maximised by lower-than-average contributions, spurred by the hope that others will compensate to reach the target^13^.

In contrast to previous work, we have here assembled 15 groups of 18 players each where the groups are sub-divided into 6 ‘countries' of 3 players each who elect, re-elect or vote out their representative for the 6 representatives' ‘summit'. For control, we have assembled 15 groups of 6 players each (as in ref. 14) and 15 groups of 18 players each. In 3 consecutive collective-risk games with 10 rounds each, each player in the 6-players and 18-players treatments contributes from her initial endowment of €40; in the 6-representatives treatment, each representative contributes from the combined endowments (€120) of her watching country mates and on their behalf (Fig. 1; see Methods). The target sum that must be collected by each group to prevent simulated dangerous climate change is €120 in the 6-players treatment and €360 both in the 18-players and the 6-representatives treatments.

We find that selfish players are preferentially elected and are hence found more frequently in the six-representatives treatment than in the other two treatments. Across all treatments, we identify the selfish players as extortioners. We develop a mathematical model and confirm its prediction that the extortioners' steadfast strategies enforce cooperation from fair players who finally compensate almost completely the deficit caused by the extortionate co-players.

## Results

### Simulated dangerous climate change

In the first game of the 18-players treatment and of the 6-representatives treatment, only 33% of the groups reach the target sum. By contrast, groups in the six-players treatment are almost twice as likely to collect sufficient contributions in the first game, with 60% of the groups reaching the target sum (Fig. 2a–c), similar to a previous study^14^. The percentage of groups reaching the target sum increases towards game 3 in the six-players and the six-representatives treatment, but the increase is not statistically significant. In game 3, the groups in the 18-players treatment are the least successful (Fig. 2a–c), but again differences are not statistically significant.

The total sums contributed per group do not differ among treatments in games 2 and 3 (Fig. 2e,f). In game 1, the six representatives contribute less than the six players (*P*=0.019, *z=*−2.341, *n*_*1*_=*n*_*2*_=15 groups, Mann–Whitney *U*-test, two-tailed; we use two-tailed tests throughout, with the group of six or 18 players as our statistical unit if not stated otherwise). Because in game 1, representatives are randomly picked from the group (see methods), the only difference between the two treatments is that representatives are contributing on behalf of their observing group. In such situations, representatives may have a more competitive mindset^7^, which would explain why groups in the six-representatives treatment reach the target less often. Total contributions show a small increasing trend from the first to the third game in all treatments (Fig. 2e,f), but the differences are statistically significant only between games 1 and 2 in the six-representatives treatment (*P*=0.026, *z=*−2.230, *n*=15, Wilcoxon signed-rank matched pairs test). Summed up over all three games per group, contributions relative to the target sum are lowest in the six-representatives treatment, significantly lower than in the six-players treatment (*P*=0.0061, *z=*−2.742, *n*_1_=*n*_2_=15, Mann–Whitney *U*-test).

### Fair and selfish players

For the group to reach the target sum, each player must on average contribute half of her total endowment—the ‘fair share' of €20 (€60 per representative in the six-representatives treatment). Thus, whenever the target sum is not reached, one or several players must have contributed less than their fair share. We call these ‘selfish players' to distinguish them from the ‘fair players' who give at least their fair share. The percentage of selfish players is highest in the 6-representatives treatment (Fig. 3a), higher than in the 6-players treatment (*P*=0.01, *z=*−2.559, *n*_1_=*n*_2_=15, Mann–Whitney *U*-test) and almost significantly higher than in the 18-players treatment (*P*=0.06, *z=*−1.862, *n*_1_=*n*_2_=15, Mann–Whitney *U*-test). The average contribution of a selfish player (relative to the fair-share contribution) is lower in the 18-players treatment than in both the 6-players (*P*=0.02, *z=*−2.302, *n*_1_=*n*_2_=15, Mann–Whitney *U*-test; Fig. 3b) and the 6-representatives treatment (*P*=0.006, *z=*−2.739, *n*_1_=*n*_2_=15, Mann–Whitney *U*-test) (Fig. 3b). Over all three games, the net payoff (including trials where the group fails to collect the target sum and loses all remaining money) is higher for selfish than for fair players (Fig. 3c). Selfish players achieve a higher net payoff in the 6-players treatment, compared with both the 18-players treatment (*P*=0.024, *z=*−2.261, *n*_1_=*n*_2_=15, Mann–Whitney *U*-test) and the 6-representatives treatment (*P*=0.020, *z=*−2.325, *n*_1_=*n*_2_=15, Mann–Whitney *U*-test, shown per represented player; Fig. 3c).

Using a classification of players in a social dilemma proposed by Fischbacher and Gächter^19^, the selfish representatives might be ‘pessimistic conditional cooperators' who dislike that others contribute less than their fair share and thus stop contributing. However, all selfish representatives contribute more in the end than in the beginning (*P*=0.0002, linear regression of contribution per selfish representative per group on rounds 1–10, analysed for game 3) and resemble ‘imperfect conditional cooperators'^19^. By increasing their contribution during the 10 rounds as do fair representatives (*P*=0.002), the selfish players help reaching the target, though they contribute much less than fair representatives.

### Voters choose selfish representatives

After both games 1 and 2, representatives can be either re-elected or voted out. After game 1, those representatives who are re-elected have contributed significantly less in game 1 than those who are voted out (Fig. 4a) (*P*=0.01, *z=*−2.587, *n*=15, Wilcoxon signed-rank matched pairs test). While this is not the case after game 2, we still find a tendency that selfish representatives are preferentially re-elected, based on their past contributions. In addition, before each election the players formulate election pledges specifying their contribution strategy if elected. The percentage of selfish pledges (see Methods) is higher among the 6 elected representatives than among all 18 players of that treatment (Fig. 4b), although significantly so only after game 2 (*P*=0.0071, *z=*−2.692, *n*=11, Wilcoxon signed-rank matched pairs test). Thus, representatives who act selfishly in game 1 are preferentially re-elected, and players who pledge to be selfish are preferentially elected after game 2.

Players classified as selfish according to their election pledges vote in 71.3% for classified selfish players and in 10.1% for classified fair representatives. Players classified as fair vote in 78.9% for classified fair players and in 14.6% for classified selfish players (the complement missing from 100% is due to players that could not be classified as either selfish or fair). Hence, selfish players want selfish representatives, and fair players want fair representatives.

Representatives who have pledged to be selfish contribute less in the following game than those who have pledged to be fair (Fig. 4c; after game 1: *P*=0.007, *z=*−2.692, *n*=10; after game 2: *P*=0.0051, *z=*−2.803, *n*=10, Wilcoxon signed-rank matched pairs test). Thus, players fulfil their pledges when acting as representatives.

### Identification of selfish players as extortioners

Theorists have predicted for a long time that cooperative and fair strategies such as Tit-for-Tat would eventually succeed in social dilemmas^20^,^21^,^22^,^23^. Why then would subjects vote for representatives who mainly pursue the success of their own subgroup while disregarding the risks for the whole community? We hypothesize that the election procedure would favour representatives who motivate the other subgroups' representatives to reach the target, but at the same time ensure that the own subgroup contributes less than other subgroups. Individuals would like their representatives to be steadfast and to convince the other subgroups' representatives to compensate for any missing contributions. Such behaviour is reminiscent of the recently discovered class of extortionate ZD strategies for the repeated prisoner's dilemma^24^,^25^,^26^,^27^,^28^,^29^,^30^, where extortionate players incentivize their opponents to cooperate although they themselves are not fully cooperative. In pairwise encounters, these extortionate players cannot be beaten by any other strategy, and they are predicted to perform well among adaptive co-players^24^,^25^,^27^,^29^. In the Methods section, we extend the theory of ZD strategies to the collective-risk social dilemma, and we prove that also in our experiment players may adopt extortionate strategies. Such players exhibit the following three characteristics: (i) Extortioners gain higher payoffs than their co-players by contributing less towards the climate account; that is, if *x*_*i*_ is the total contributions of an extortioner, and if *x*_*−i*_ is the average contribution of the other group members, then


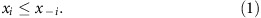


(ii) Extortioners persuade their co-players to make up for the missing contributions; that is, the collective best response for the remaining *N*−1 group members is to choose *x*_*−i*_ such that the group reaches the target sum *T*,


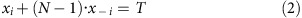


(iii) Extortioners are consistent, meaning that the properties (i) and (ii) are not only satisfied in one particular instance of the game, but in every game the player participates in. We now test whether the selfish players in our experiment meet these three criteria.

Because we find both fair and selfish players in all three treatments, we perform a proof-of-principle with players of all treatments combined. To keep the group as statistical unit, we enter contribution averaged over all fair players of each group; contributions of representatives are divided by 3 to be comparable ‘per player' to the other treatments. The contribution per fair player increases over the three games (Fig 5a; *P*=0.0057, F_2,130_=5.3788, generalized linear model (GLM) with family=Gaussian). By contrast, the contribution per selfish player does not increase significantly (*P*=0.66, F_2,131_=0.4163, GLM). We find a significant interaction between fair and selfish players' contributions over the three games (*P*=0.032, F_2,261_=3.4798, GLM). Over the three games, as the contributions of fair players increase, so does the payoff of both fair players (*P*=0.010, F_2,132_=4.7574, GLM, with family=gamma) and selfish players (*P*=0.015, F_2,132_=4.339, GLM, with family=gamma; Fig. 5b). In each game, selfish players gain more than fair players; the difference increases from game 1 to game 3 (Fig. 5c) (*P*=0.046, *z=*−1.995, *n=*45, Wilcoxon matched pairs signed ranks test).

To test whether other group members are willing to compensate for missing contributions, we compare the contribution deficit of all selfish players in a group (the sum of all their negative deviations from the fair share) with the contribution surplus of all fair players (the sum of positive deviations of all the fair players; Fig. 6). For example, in game 1 in the six-players treatment, the dot most to the left (Fig. 6a) shows a group where the five selfish players contribute only €80 instead of the fair-share contribution of €100. The single fair player of that group contributes €22, €2 more than her fair share but not enough to compensate for the deficit of €20 caused by the selfish players. Hence the group misses the target sum of €120, and everybody loses the money not invested with 90% probability. As another example, the leftmost dot of those exactly on the red line depicts a group where the three selfish players invest €44 instead of €60, causing a deficit of €16, which is exactly compensated by the three remaining fair players. Thus the group meets the target of €120, but the selfish players receive a higher payoff than the fair players.

If selfish players were indeed able to persuade the remaining group members to compensate for missing contributions, we would expect the regression lines in Fig. 6 to have a significantly negative slope and to be close to the red lines marking exact (hypothetical) compensation. We see this compensation in the six-players treatment in game 2 (Fig. 6b, simple regression, F-test=36.257, degree of freedom (DF)=1, *P*=0.0001) and in game 3 (Fig. 6c, simple regression, F-test=26.204, DF=1, *P*=0.0002) and in the six-representatives treatment in game 3 (Fig. 6f, simple regression, F-test=17.286, DF=1, *P*=0.0011). By contrast, we find no significant compensation in the 18-players treatment.

In the 6-players treatment, fair players compensate or overcompensate the selfish players' deficit in 9 groups in games 1 and 2 (Fig. 6a,b) and in 13 groups in game 3 (Fig. 6c). In the 18-players treatment, fair players compensate or overcompensate the selfish players' deficit in 5 groups in game 1 (Fig. 6g) and in 7 groups in games 2 and 3 (Fig. 6h,i). In the 6-representatives treatment, the deficit of the selfish players is only compensated in 4 groups in game 1 (Fig. 6d) but in 9 groups in game 2 (Fig. 6e) and in 10 groups in game 3 (Fig. 6f). Over all treatments and games, selfish players or selfish representatives successfully drive their fair counterparts to compensation in 73 out of 135 individual games (54%). Moreover, groups become increasingly successful in reaching the target, improving from game 1 (40%) to game 2 (56%) and game 3 (67%). Because only fair players raise their contributions over the three games but not selfish players (see Fig. 5a), these results suggest that a considerable fraction of fair players learn to become even more cooperative in response to extortioners. The learning effect is demonstrated by the observation that the contribution per fair representative has no relation to the number of selfish representatives per group in game 1 but correlates significantly in game 3 (Supplementary Fig. 2).

Players behave consistently across the 3 games in the 6-players and the 18-players treatments, as witnessed by significant positive correlation of the contributions (see Supplementary Information for detailed analysis). For the six-representatives treatment, we have analysed the behaviour of representatives after being re-elected. In 34 out of the 42 cases in which a selfish representative is re-elected, the representative remains selfish in the next game (*P*=0.005, Fisher's exact test, two-tailed compared with 50%). Overall, we have thus established that selfish players gain much higher payoffs (Fig. 5); they are often successful in persuading their fair co-players to compensate for missing contributions (Fig. 6); and they are consistent across different games. Thus, selfish players show all three characteristics of extortionate behaviour.

## Discussion

We have introduced into the collective-risk social dilemma the innovation that contributions into the climate account are decided on not by individual players but by representatives (six-representatives treatment). For control, we have assembled groups of 6 players and 18 players in 2 further treatments. We find selfish players in all treatments, but their concentration is highest in the 6-representatives treatment. Selfish representatives are preferentially elected or re-elected if they either contribute less than the fair share or pledge to do so. Having to cater to their electorates' preferences thus has the adverse effect that representatives risk losing the climate game to win elections. As a consequence, groups in the six-representatives treatment contribute less than groups in the six-players treatment (relative to the required target sum), and they receive lower average payoffs. On the other hand, in games 2 and 3 the groups tend to reach the target sum more often in the 6-representatives than in the 18-players treatment. While fair representatives compensate for missing contributions in game 3, 18 players do not achieve that compensation. Thus, our representatives tend to be more successful in preventing simulated dangerous climate change than 18 players deciding themselves. We speculate that this ‘representatives' advantage' is much greater with much larger groups such as real countries.

The psychological consequences of acting as a representative of a group have been characterised as evoking both more competitive interaction goals and more competitive expectations of others^7^. A representative is faced with a powerful responsibility to provide good outcomes for her constituency and may face strong pressures by being monitored and evaluated^7^. The mindset that is activated by the role of representative shows up clearly in our experiments when we compare the behaviour of six players randomly selected to decide for themselves with the behaviour of six representatives randomly selected to decide for their group. Otherwise the players find themselves in exactly the same situation in both cases. As psychology predicts^7^, the six players' groups are twice as successful as the six-representatives' groups in reaching the target sum. When voting, players preferentially choose representatives who either have displayed a competitive mindset as former representatives or have pledged to do so if elected.

In our experiments, selfish behaviour pays off only if others compensate any missing contributions. Selfish subjects apply an implicit form of extortion^24^—they contribute less than is needed on average, but in a way that makes it optimal for their peers to become even more cooperative. The effect of extortion in our experiments differs from that in the repeated prisoner's dilemma, in which subjects strongly oppose exploitation^31^. Here subjects in the six-players and six-representatives treatments eventually accept extortion up to a certain degree, especially in game 3, in which subjects have already gained some experience. We speculate that the higher tolerance towards extortioners in our experiment is due to the higher stakes involved—resisting extortion comes relatively cheap in the prisoner's dilemma, but it endangers the entire payoff in the collective-risk game. Only in the 18-players treatment was extortion unsuccessful in persuading others to cooperate, presumably because in larger groups it becomes more difficult to induce individuals to behave in a desired way.

Our identification of extortionate behaviour in the collective-risk social dilemma suggests two counteracting major effects when, with all due caution, we try to interpret the social dynamics of climate summits with our results in mind. On the one hand, the competitive advantage of selfish players in getting elected or re-elected appears to work against reaching a collective target such as preventing dangerous climate change—there might not be enough fair representatives around to support the target. On the other hand, selfish players, who are ubiquitous and show up in all but 1 of the 135 individual collective-risk games, consistently act as extortioners. Their steadfast strategies enhance the already-existing willingness of our fair players to contribute towards reaching the collective target. If we compare extortionate to hypothetical non-extortionate selfish players, we conclude—with more than just a hint of Machiavellian thinking—that extortion benefits the prevention of dangerous climate change.

## Methods

### Experimental procedures

A total of 630 undergraduate students from the Universities of Bonn, Hamburg, Göttingen, Kiel and Münster voluntarily participated in 45 experimental sessions with either 18 or 6 subjects each in a computerized experiment (for example, ref. 32). The subjects were separated by opaque partitions and each had a computer, on which they received the instructions for the experiment and with which they communicated their decisions. Throughout the whole experiment, subjects were anonymous, and they made their decisions under a neutral pseudonym.

**Figure 1:**
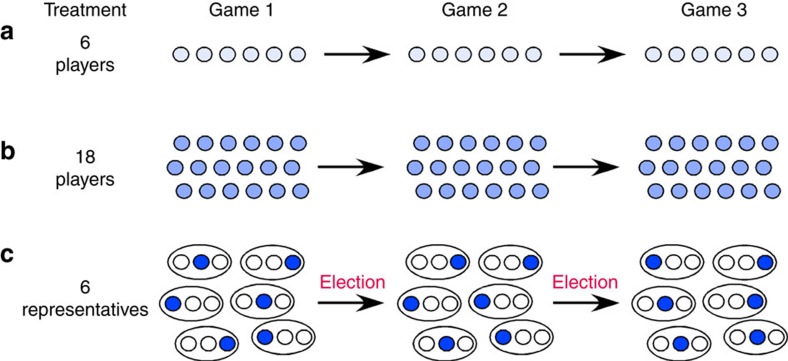
Design of the three treatments. (**a**) The 6-players treatment, (**b**) the 18-players treatment, (**c**) the 6-representatives treatment. Each game consists of 10 rounds, during which players need to raise sufficient contributions to reach a specified target sum. Games 2 and 3 are replicates of game 1. The players remain the same in the 6-players and the 18-players treatment. In the 6-representatives treatment, representatives are randomly picked in game 1 and re-elected or voted out for games 2 and 3. Re-election of a representative may depend on the representatives' performance in previous games. In addition, except for the first four groups, after games 1 and 2 all players in the 6-representatives treatment are asked to write non-binding pledges about how they would contribute if elected. Players are only informed about the pledges of members of their own subgroup.

**Figure 2:**
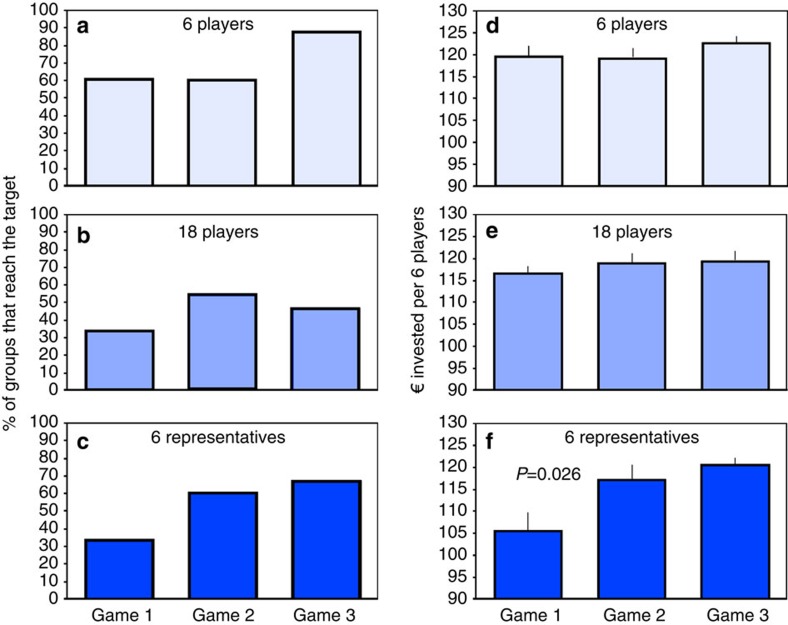
Group success in reaching the target sum (left) and group investments (right). (**a**,**d**) Six-players treatment; (**b**,**e**) 18-players treatment; (**c**,**f**) 6-representatives treatment. In **f**, the sum invested is divided by 3 to allow comparison among treatments. Means±s.e.m. of 15 groups per game and treatment are shown. See text for statistics.

**Figure 3:**
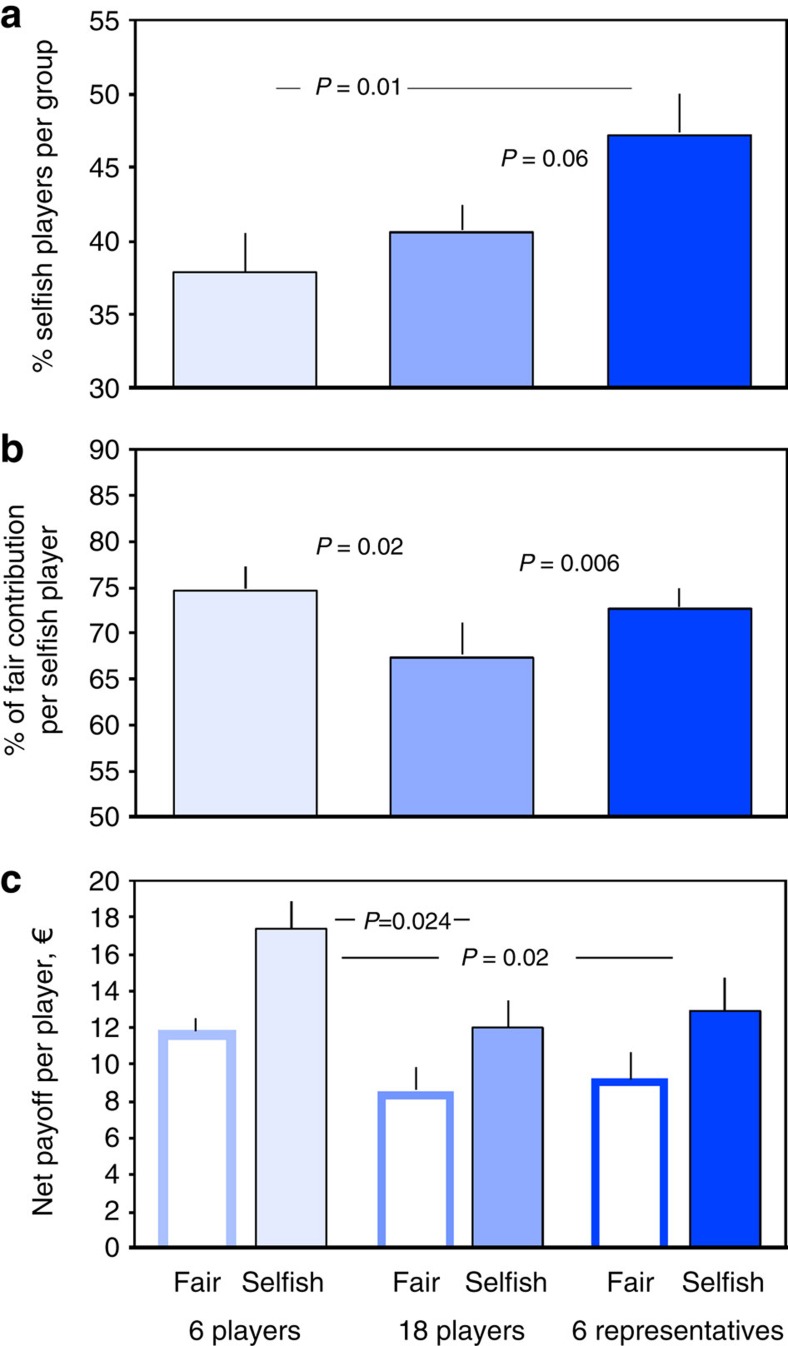
Fair and selfish strategies. (**a**) The percentage of selfish players per group, (**b**) the average contribution of a selfish player (relative to the fair-share contribution), (**c**) the net payoff per fair and selfish player. Means±s.e.m. of 15 groups per treatment are shown. See text for statistics.

**Figure 4:**
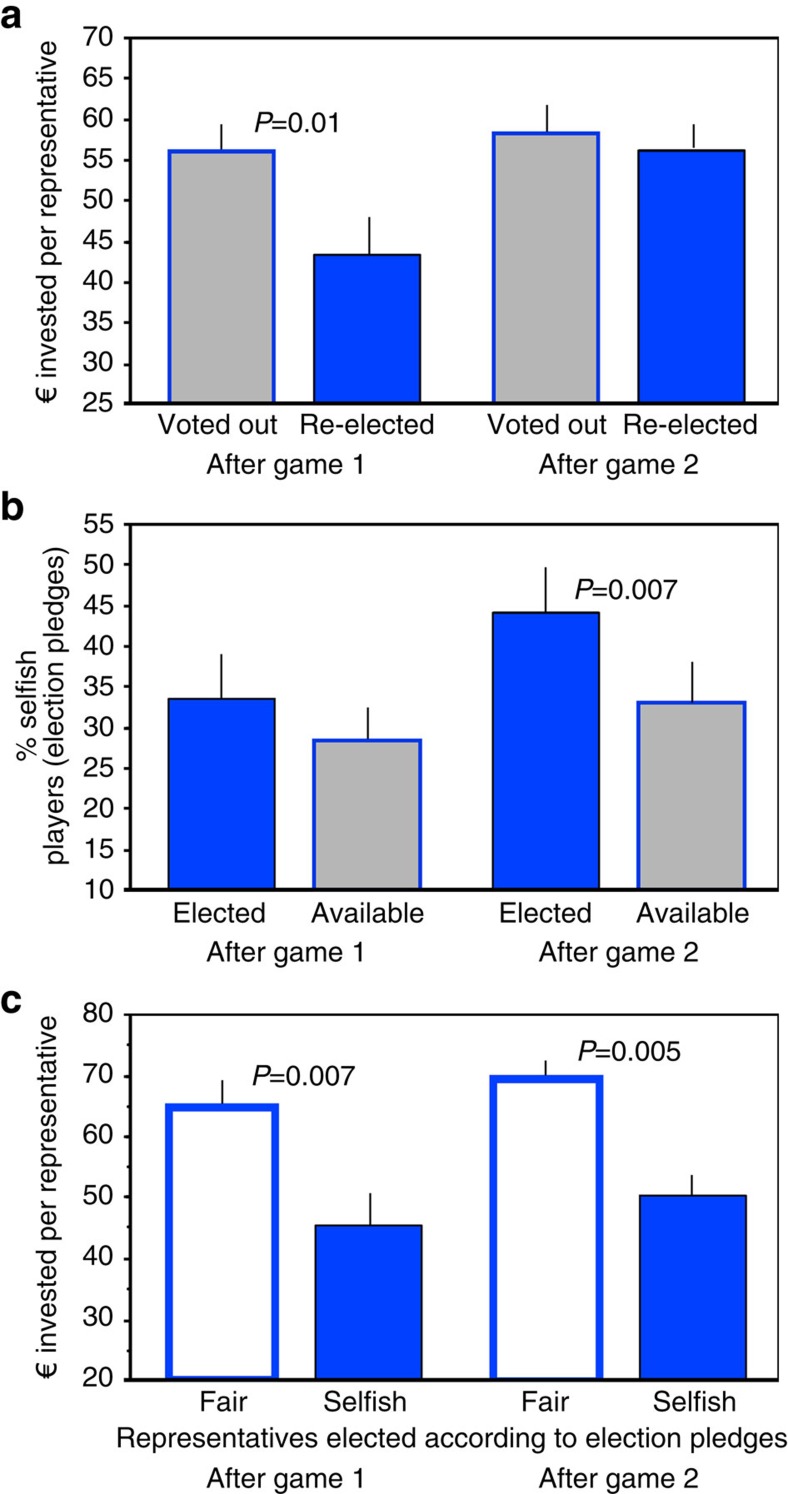
Voting success and behaviour of selfish and fair representatives in the six-representatives treatment. (**a**) Previous investment of representatives who are either voted out or re-elected, (**b**) percentage of selfish players, according to their election pledges, available and elected, (**c**) future fulfilment of election pledges by selfish and fair players. Means±s.e.m. groups are shown, for 15 groups in **a** and 11groups in **b** and 10 groups in **c**. See text for statistics.

**Figure 5:**
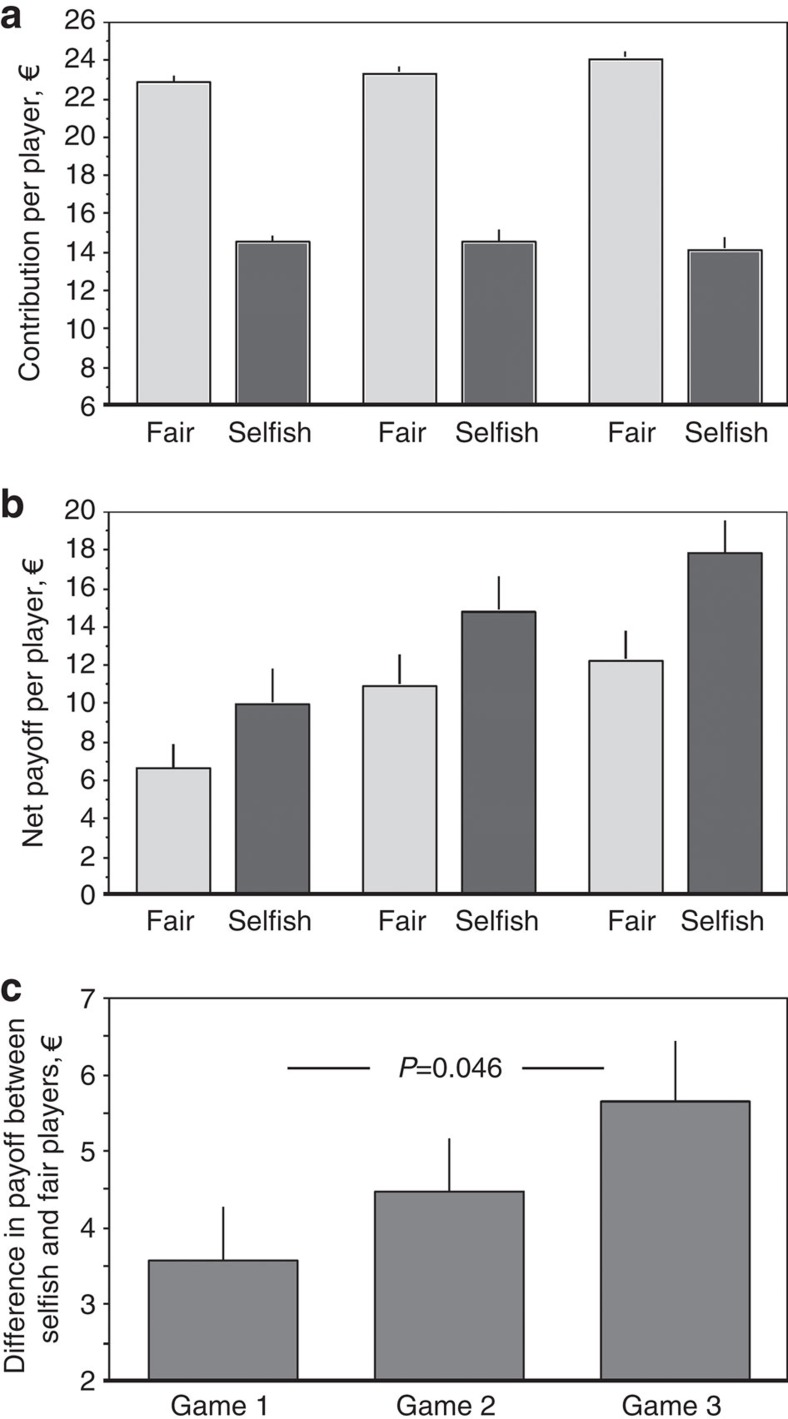
Comparison of contributions and payoffs for fair players and selfish players across all three games. (**a**) Contribution of fair and selfish players; (**b**) net payoff of selfish and fair players; (**c**) difference in payoff between fair and selfish players. We enter contributions averaged over both all fair and all selfish players of each group. Contributions of representatives are divided by 3 to be comparable to other treatments. See text for statistics.

**Figure 6:**
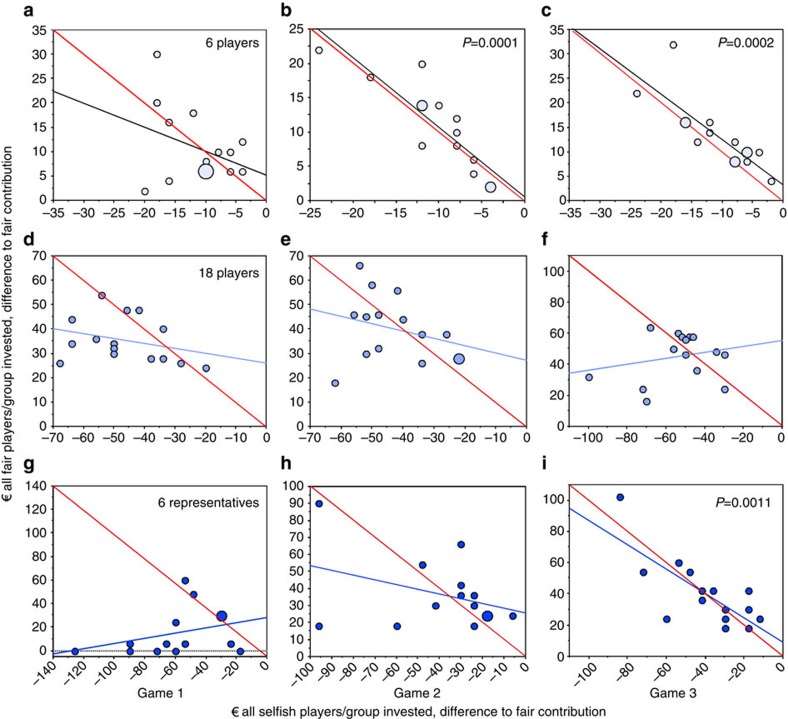
Fair players' compensation of their selfish players' deficit. (**a**–**c**) Six-players treatment; (**d**–**f**) 18 players treatment; (**g**–**i**) 6-representatives treatment. Each dot represents a group; larger dots show overlaid results from two or three groups. Black and blue lines depict simple regressions. The red lines depict all combinations of hypothetical contributions in which fair players exactly compensate for the deficit caused by all selfish players of that group. Thus, dots on or above the red line correspond to groups that reach the target sum. See text for statistics.

There are three treatments (Fig. 1). For each treatment, we had 15 groups of subjects interacting in a variant of the ‘collective-risk social dilemma' game^14^: subjects received an initial endowment, and they were asked, in each of 10 rounds, to contribute money from this endowment into a ‘climate account'. At the end of round 10, the game software checked whether total contributions of all group members matched (or exceeded) a previously specified target sum. If that was the case, subjects received their remaining endowment in cash (in a way that maintained the subjects' anonymity). If the collective target was not reached, subjects lost their remaining endowment with 90% probability. Each game was repeated twice, such that every group played three games with all players keeping their pseudonyms (each time with a new endowment).

In the 6-players and the 18-players treatments, groups consisted of 6 and 18 subjects, respectively, and each subject had an initial endowment of €40. In each round, subjects could choose whether to contribute €0, €2 or €4 to the climate account, and the decisions of all subjects were shown to all subjects after each round. The target was reached if on average subjects contributed half their endowment (the target sum was €120 in the 6-players treatment, and it was €360 in the 18-players treatment).

In the 6-representatives treatment, groups of 18 subjects were sub-divided in 6 ‘countries' of 3 players. For game 1, the computer randomly determined six representatives, one from each country. Only the representatives were able to contribute money to the group's climate account: they had 3 times €40 at their disposal, for investing €0, €6 or €12 in each of 10 rounds. The decisions of all representatives were shown to all 18 subjects after each round. The target was to collect at least €360 in donations (€60 per representative or €20 per subject). If the target was reached, subjects received a third of their country's remaining endowment.

After games 1 and 2, the three subjects of each country could re-elect the previous representative or vote her out and elect a different member of their country with a majority vote. Except for the first four groups of this treatment, subjects could compose both after game 1 and game 2 election pledges of up to 500 characters on their laptop that could be seen only by the 3 subjects of a country. The pledges described how the person would decide if elected. We have blindly classified all pledges; those that promise to contribute ‘less than the others', or ‘less than the fair share' have been classified as ‘selfish' and the others as ‘fair'. When making the voting decision, each subject knew the observed decisions of her previous representative and those of the other representatives, and saw the three election pledges within her country (each with the respective pseudonym and a button for voting). In cases with no majority vote, the computer decided randomly for the next representative (in 9% of cases).

Subjects knew that the total sum of money in the climate account, accumulated from all participating groups, would be used to publish a press advertisement on climate protection in a daily German newspaper simultaneously with the publication of the present study. However, they received the ‘little information' version from ref. 32 to explain the climate account, so that we could expect very weak motivation to invest in publishing the advertisement *per se*.

### Theoretical model

Press and Dyson^24^ describe a class of so-called ZD strategies for the repeated prisoner's dilemma, and they demonstrate that a subset of ZD strategies can be used to extort opponents. However, the collective-risk dilemma game used in our experiment is not a repeated two-player game. Herein, we thus extend the theory of ZD strategies to collective-risk dilemmas. As an application, we show the existence of extortionate strategies. Such strategies ensure that (i) a player gets at least the average payoff of the co-players; (ii) the collective best reply for the remaining group members is to reach the target; and (iii) the properties (i) and (ii) hold in any game the player participates in.

To this end, we consider a group of *N* individuals, with each group member having an initial endowment of *E*. The group engages in a collective-risk dilemma^12^: in each of *R* rounds, players can decide how much they want to contribute towards a common pool. We denote player *i*'s contribution in round *r* by *x*_*i*_(*r*), and we assume that the minimum contribution per round is 0, whereas the maximum contribution is *x*_max_=*E*/*R*. To calculate the total contributions *x*_*i*_ of player *i*, we sum up over all rounds, *x*_*i*_=∑*x*_*i*_(*r*). The group's total contributions *x* are obtained by summing up over all individual contributions, *x*=∑*x*_*i*_. Payoffs for the collective-risk dilemma are defined as follows: if total contributions after round *R* exceed a threshold *T*, then all players receive their remaining endowment; that is, if *x*≥*T*, then player *i*'s expected payoff is *E*−*x*_*i*_. Otherwise, if total contributions are below the threshold, all players risk losing their remaining endowment with some probability *p*>0, and player *i*'s expected payoff becomes (1−*p*) (*E*−*x*_*i*_). Supplementary Table 1 gives a summary of all used variables.

In the experiment, players had to choose between three possible contribution levels in a given round, but for the model we assume for simplicity that players can contribute any amount *x*_*i*_(*r*)∈[0, *x*_max_]. We note that the definition of ZD strategies given below can be extended to the case of discrete contribution levels. To achieve an arbitrary contribution level *y*∈[0, *x*_max_], player *i* would need to randomize between the given discrete contribution levels such that the expected value satisfies *E*[*x*_*i*_(*r*)]=*y*. Similar to Tit-for-Tat-like strategies in the Prisoner's Dilemma, we define ZD strategies in the collective-risk dilemma as behaviours that condition their contribution in the next round on the co-players' contributions in the previous round:

*Definition (ZD strategies)*. Player *i* applies a ZD strategy for the collective-risk dilemma if *i*'s contributions *x*_*i*_(*r*) in every round *r* satisfy


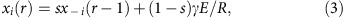


where *x*_*−i*_(*r*−1) is the average contribution of the other group members in the previous round, with *x*_*−i*_(0):=0, and *s* and *γ* are parameters that can be chosen by player *i*.

The parameter *s* is a measure for how a player reacts to the co-players' contributions of the previous round. The parameter *γ*, on the other hand, determines a player's baseline contribution level. These two parameters cannot be chosen arbitrarily—since player *i*'s contribution needs to be in the interval [0, *E/R*], the two parameters need to satisfy


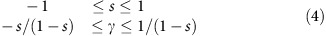


It is the following property that makes ZD strategies interesting.

*Proposition 1 (properties of ZD strategies)*. Suppose player *i* applies a ZD strategy with parameters *s* and *γ*.

1. If *x*_*i*_ denotes the total contributions of player *i*, and if *x*_*−i*_ denotes the average total contribution of *i*'s co-players, then


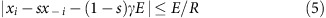


2. Similarly, if *π*_*i*_ and *π*_*−i*_ denote the corresponding realized payoffs, then payoffs either satisfy *π*_*i*_=*π*_*−i*_=0 (if the group fails to reach the threshold and dangerous climate change occurs), or


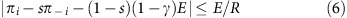


*Proof*

1. By summing up Equation (3) over all rounds 1≤*r*≤*R*, we obtain


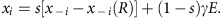


As a consequence,


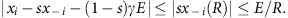


2. In case players do not lose their remaining endowment, Equation (6) follows directly from Equation (5) because *π*_*i*_=*E*–*x*_*i*_ and *π*_*−i*_=*E*−*x*_*−i*_.

For a collective-risk dilemma with sufficiently many rounds *R*, Proposition 1 thus implies that *x*_*i*_≈*sx*_*−i*_+*γ*(1−*s*)*E*. That is, there is a linear relationship between the total contributions of player *i*, and the total contributions of *i*'s co-players. Similarly, it follows for the realized payoffs that either *π*_*i*_=*π*_*−i*_=0 or *π*_*i*_≈*sπ*_*−i*_+(1−*γ*)(1−*s*)*E*. Therefore, unless payoffs are zero, there is also a linear relationship between the players' realized payoffs. This property makes strategies having the form of Equation (3) analogous to the ZD strategies described for the repeated prisoner's dilemma^24^. It is important to note that the above Proposition makes no restrictions on the strategies of *i*'s co-players—the stated results hold no matter what the other group members do. As a particular instance of ZD strategies, let us consider the following special case.

*Definition (extortionate ZD strategies)*. A player applies an extortionate ZD if the parameters *s* and *γ* are chosen such that


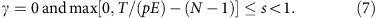


If some player *i* applies such an extortionate strategy, it follows from Proposition 1 that approximately *i*'s total contribution only make up a fraction of the average contribution of the other group members, since *x*_*i*_≈*sx*_*−i*_ (Supplementary Fig. 1 gives an illustration).

The following Proposition shows that the name ‘extortionate ZD strategy' is justified: players with such a strategy show the typical characteristics of extortionate behaviour.

*Proposition 2 (properties of extortionate ZD strategies)*. Suppose player *i* applies an extortionate ZD strategy. Then, irrespective of the strategies applied by the other group members (that is, in any game player *i* participates in),

1. Player *i*'s realized payoff is never below the mean payoff of the other group members, *π*_*i*_≥*π*_*−i*_.

2. The collective best reply for the remaining group members is to reach the threshold *T*. In that case, player *i*'s payoff is strictly better than average, *π*_*i*_>*π*_*−i*_.

*Proof.* Because a player with an extortionate ZD strategy contributes strictly less than average, *x*_*i*_<*x*_*−i*_, it follows that either *π*_*i*_=*π*_*−i*_=0 (if the group misses the target and players lose their remaining endowment) or *π*_*i*_>*π*_*−i*_ (otherwise). Moreover, for the other group members, it is collectively optimal to reach the target: by contributing nothing, their expected payoff becomes (1−*p*)*E*, whereas if they make the minimum contribution (in the first *R*−1 rounds) such that total contributions reach the target, then their payoff is *E*−*T*/(*N*−1+*s*). Because *s*≥*T*/(*pE*)−(*N*−1), reaching the target is a collective best reply.

Proposition 2 is a proof-of-principle: there are strategies for the collective-risk dilemma that allow a player to extort the other group members. We note that the set of all extortionate strategies will typically be considerably bigger than the set of all extortionate ZD strategies. When we analyse experimental data, we therefore do not specifically look for strategies that have the functional form described in Equations (3) and (7); we rather look for all possible strategies that indicate extortionate behaviour (that is, we look whether players satisfy the conditions (i)–(iii) defined in the main text).

## Additional information

**How to cite this article:** Milinski, M. *et al.* Humans choose representatives who enforce cooperation in social dilemmas through extortion. *Nat. Commun.* 7:10915 doi: 10.1038/ncomms10915 (2016).

## Supplementary Material

Supplementary Information Supplementary Figures 1-2, Supplementary Table 1 and Supplementary Methods.
