# Supplementary material for: Humans choose representatives who enforce cooperation in social dilemmas through extortion
Source: Nat Commun. 2016 Mar 7;7:10915. doi: 10.1038/ncomms10915 (PMC4786683; doi:10.1038/ncomms10915)
Supplement: Supplementary Information — Supplementary Figures 1-2, Supplementary Table 1 and Supplementary Methods. [file ncomms10915-s1.pdf]

Supplementary Information

Supplementary Figures

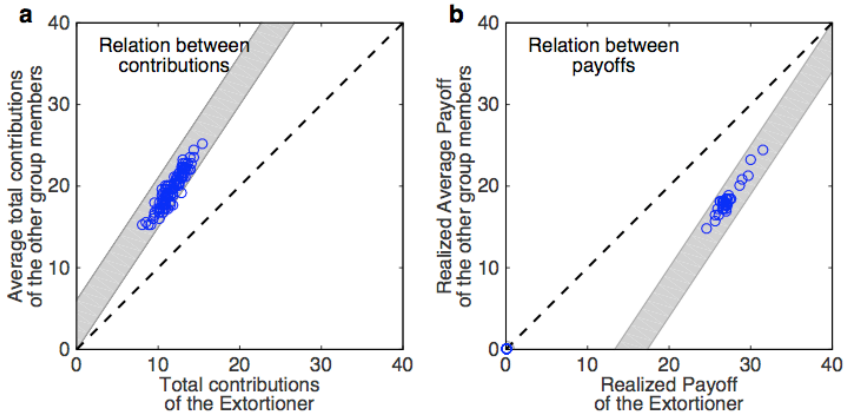

Supplementary Figure 1 | Simulated consequences of extortionate behaviour. a, the relationship between the players' contributions, and **b**, the relationship between payoffs if one group member adopts an extortionate strategy. We have simulated the outcome of 100 groups that contain a player with an extortionate ZD strategy (with parameter $s = 2/3$), interacting in a collective-risk dilemma with random co-players (each group is depicted by a blue dot). The grey-shaded areas give the predicted regions for contributions and payoffs according to Proposition 1. Extortioners contribute systematically less than the other group members (i.e., in **a**, all 100 dots are above the dashed diagonal). As a result, extortioners either get the same payoff as the co-players (in case they all get 0 because the group failed to avoid dangerous climate change), or the extortioner gets a higher payoff than the co-players (i.e., in **b**, all blue dots are on or below the dashed diagonal). For the simulations, we have taken the parameters of the 6-players treatment (with the only exception that contributions are allowed to be continuous).

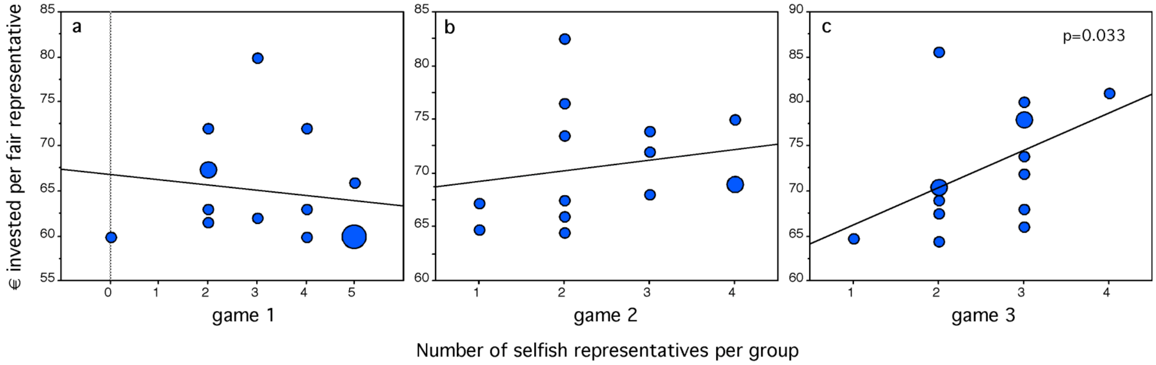

Supplementary Figure 2 | Fair representatives compensate until game 3 depending on the number of extortioners per group, a, in game 1, **b**, in game 2, **c**, in game 3 (p = 0.033, Spearman rho = 0.569, z = 2.128, n = 15). Larger dots show overlaid dots of 2 or 3 groups.

Supplementary Tables

Variable name	Description	Value in the experiment 6 players	18 players	6 representatives
N	Number of (active) players	6	18	6
R	Number of rounds	10	10	10
E	Initial endowment of a player	€ 40	€ 40	3 x € 40
T	Target sum for total contributions	€ 120	€ 360	€ 360
p	Probability to lose remaining endowment	90 %	90 %	90 %
$x_i(r)$	Contributions of player i in round r	€0, €2, or €4	€0, €2, or €4	€0, €6, or €12

Supplementary Table 1 | Overview for the used variables for the theoretical model. The table shows all parameters that were used for the theoretical model, and gives the corresponding parameter values in each of the three experimental treatments.

Supplementary Methods

1. Further experimental results

Players are consistent in their strategy over consecutive games. Both fair and selfish players are consistent in their decisions between games in all three treatments. In the 6-players treatment we find between individual contributions in games 1 and 2 a positive correlation; mean Corr. Coeff. ± s.e.m. over all groups is 0.474±0.117 (different from 0, $p = 0.005$, $z = -2.783$, $n = 15$, Wilcoxon one-sample test), and between games 2 and 3 a positive correlation; mean Corr. Coeff. ± s.e.m. over all groups is 0.50 ± 0.13 (different from 0, $p = 0.013$, $z = -2.499$, $n = 15$).

Players are consistent in their strategy over consecutive games also in the 18-players treatment from game 1 to game 2; positive correlation, mean Corr. Coeff. ± s.e.m. over all groups is 0.57 ± 0.056 (different from 0, $p = 0.0007$, $z = -3.408$, $n = 15$, Wilcoxon one-sample test), and from game 2 to game 3, positive correlation, mean Corr. Coeff. ± s.e.m. over all groups 0.639 ± 0.054 (different from 0, $p = 0.0007$, $z = -3.408$, $n = 15$).

In the 6-representatives treatment, we can compare only representatives who have been re-elected after game 1, rho=0.466 ($p = 0.0094$, $z = -2.597$, $n = 32$ players, Spearman Corr. Coef.) and after game 2, rho=0.702 ($p = 0.0001$, $z = 4.548$, $n = 44$ players). Here we cannot test for correlations within groups, because in some cases only one representative has been re-elected. Overall, both fair and selfish players are consistent in their decisions in all three treatments.

2. Advertisement in daily newspapers of those University cities where experiments were performed

Text:

Professor Jochem Marotzke, Direktor am Max-Planck-Institut für Meteorologie:
„Der Mensch hat das globale Klima bereits nachweislich geändert, und weitere, wesentlich größere Änderungen sind für dieses Jahrhundert zu erwarten. Der Ausstoß von CO_2 und anderen Treibhausgasen wird die globalen Erwärmung weiter verstärken. Als Folge müssen wir mit häufigerem Auftreten von Klima- und Wetterextremen rechnen. Hitzewellen wie 2003 in Europa, mit über 15.000 Todesfällen allein in Frankreich, werden häufiger auftreten. Der Wasserkreislauf in der Atmosphäre wird sich verstärken – Trockengebiete werden trockener, feuchte Gebiete feuchter. Sowohl Dürren als auch Extremniederschläge und Überschwemmungen werden zunehmen. Der Meeresspiegel wird ansteigen, und dadurch die Gefahr extremer Sturmfluten."

„Einige künftige Klimafolgen des menschengemachten CO_2-Ausstoßes, wie eine gewisse Erwärmung und ein Anstieg des Meeresspiegels, sind bereits nicht mehr abwendbar, und die Menschheit muss sich diesen Veränderungen anpassen. Andere Folgen sind durch eine Verringerung des CO_2-Ausstoßes vielleicht abwendbar. Maßnahmen im täglichen Leben können zum Klimaschutz beitragen:

• Dreiviertel des Energieverbrauchs in Privathaushalten erfolgt durch Raumheizung. Durch eine leichte Verringerung der Raumtemperatur im Winter lässt sich viel Energie einsparen.
• Im Verkehr besteht eine Energiesparmöglichkeit durch eine stärkere Nutzung öffentlicher Verkehrsmittel statt des Privat-PKWs.
• Der verstärkte Einsatz regenerativer Energiequellen trägt zu einer Verminderung des CO_2-Ausstoßes bei."

Verantwortlich für den Text: Professor Jochem Marotzke, Max-Planck-Institut für Meteorologie, Hamburg. Das „Klimaspiel" wurde in Kooperation mit dem Max-Planck-Institut für Evolutionsbiologie in Plön durchgeführt. Diese Anzeige wurde durch Spenden finanziert.
Studierende der Universitäten Bonn, Göttingen, Hamburg, Kiel und Münster nahmen an einem „Klimaspiel teil, das die Bereitschaft der Teilnehmer erforschte, eigenes Geld für den Klimaschutz auszugeben.

Kieler Nachrichten, 12 September 2015, €2056.32
Hamburger Abendblatt, 12 September 2015, €2831.40
Westfälische Nachrichten/Münstersche Zeitung, 12 September 2015, €1257.20
Göttinger Tageblatt, 12 September 2015, €1852.20
Kölner Stadtanzeiger/Kölner Rundschau, 19 September 2015, €3614.80
Bonner Generalanzeiger, 12 September 2015, €1084.92

Total €12692.84 + 19%VAT

3. Instructions for the three treatments

(a) 6-players treatment:

Welcome to this experiment in which you can earn money!
At the start of the experiment, 40 euros will be credited to your account as your personal endowment. In the course of the game you can decide whether money from your endowment will be invested or not. At the end of the game the savings in your account will be paid cash to you. All your decisions are anonymous. To ensure this, the computer assigns a pseudonym to you, visible at the bottom left of the screen. These pseudonyms are names of moons in our solar system (Ananke, Telesto, Despina, Japetus, Kallisto or Metis). **For the experiment to be successful you are not allowed to talk to the other participants or make yourself noticeable to them in any way..**
You must confirm all information obtained during the course of the game by clicking NEXT.
After reading this text completely, please confirm by clicking NEXT.

In the course of this experiment you will play **exactly 10 climate rounds**.
In each of these rounds you can invest in an attempt to protect climate and to avoid dangerous climate change. One of the consequences of dangerous climate change will be serious economic loss, which is simulated in this game.
In each round of the game all six players will be asked simultaneously:

"How much do you want to invest in climate protection?"

(possible answers: €0, €2 or €4)

When every player has made his decision all six choices are displayed simultaneously on all six laptops. Afterwards all contributions will be credited to an account for climate protection.
Of the money that has eventually been invested in climate protection, we will place an advertisement in your local newspaper. This advert will give general information about

simple methods for climate protection - methods that everybody can implement without much effort to protect our climate and to assist in avoiding dangerous climate change. The more money we collect the larger and more conspicuous the advertisement will be. If this promotion is successful, sponsors for international advertising campaigns could be eventually mobilized.
After reading this text completely, please confirm by clicking NEXT.

Professor Jochem Marotzke, Director of the Max-Planck-Institute for Meteorology in Hamburg will provide expertise about the state of the climate for the advertising copy. He will also give some recommendations on how to abate CO_2-emissions and how the climate can be protected:
For example, energy can be saved by decreasing ambient room temperature or by the use of public transportation instead of private cars. Furthermore, the increased use of renewable energy sources contributes to greenhouse gas abatement.
After reading this text completely, please confirm by clicking NEXT

Continued: climate round

After each round the decisions of all players are displayed
Example:
Four players have decided to invest into climate protection. Two of them paid €2 and two of them paid €4.

Pseudonym	**Leda**	**Triton**	**Portia**	**Sinope**	**Carpo**	**Galatea**
Decision	**Yes**	**No**	**No**	**Yes**	**Yes**	**Yes**
Change in the player's account	**-2,00**	**0,00**	**0,00**	**-4,00**	**-2,00**	**-4,00**

In total 12 euros were paid for climate protection and thus credited to the climate

account.

After reading this text completely, please confirm by clicking NEXT

The end of the game

At the end of the game (after exactly 10 rounds) the computer compares the climate account with the threshold amount of €120. This threshold amount has to be reached to avoid dangerous climate change. It is reached if every player pays on average €2 per round for climate protection. In this case €12 per round would be paid into the climate account. If this amount has been assembled in the climate account, each player will obtain whatever is left over in his account. The money will be paid out in cash anonymously under your pseudonym. If the threshold amount has **not** been assembled in the climate account dangerous climate change occurs with a probability of **90 %**

(thus in 9 out of 10 cases)

followed by serious economic losses. **This is the probability of losing the money in your account and no group member receives a payment.**

There is a probability of **10% (thus in 1 out of 10 cases**) that you will keep the money in your account. The payment will be paid out in cash anonymously under your pseudonym. The computer decides randomly by "throwing a virtual dice" with this probability.

After reading this text completely, please confirm by clicking NEXT.

The experiment will start now

After reading this text completely, please confirm by clicking NEXT.

(b) 18-players treatment:

Welcome to this experiment in which you can earn money!

At the start of the experiment, 40 euros will be credited to your account as your personal endowment. In the course of the game you can decide whether money from your endowment will be invested or not. At the end of the game the savings in your

account will be paid cash to you. All your decisions are anonymous. To ensure this, the computer assigns a pseudonym to you, visible at the bottom left of the screen. These pseudonyms are names of moons in our solar system (Ananke, Telesto, Despina, Japetus, Kallisto etc.). **For the experiment to be successful experiment you are not allowed to talk to the other participants or make yourself noticeable to them in any way.** You must confirm all information obtained during the course of the game by clicking NEXT.
After reading this text completely, please confirm by clicking NEXT.

In the course of this experiment you will play **exactly 10 climate rounds**.
In each of these rounds you can invest in an attempt to protect climate and to avoid dangerous climate change. One of the consequences of dangerous climate change will be serious economic loss, which is simulated in this game. In each round of the game all 18 players will be asked simultaneously:

"How much do you want to invest in climate protection?"

(Possible answers: 0, 2 or 4 euros)

When each player has made his decision all 18 choices are displayed simultaneously on all 18 laptops. Afterwards all contributions will be credited to an account for climate protection.
Of the money that has eventually been invested in climate protection, we will place an advertisement in your local newspaper. This advert will give general information about simple methods for climate protection - methods that everybody can implement without much effort to protect our climate and to assist in avoiding dangerous climate change. The more money we collect the larger and more conspicuous the advertisement will be. If this promotion is successful, sponsors for international advertising campaigns could be eventually mobilized.
After reading this text completely, please confirm by clicking NEXT

Professor Jochem Marotzke, Director of the Max-Planck-Institute for Meteorology in Hamburg will provide expertise about the state of the climate for the advertisement. He will also give some recommendations on how to abate CO_2-

emissions and how the climate can be protected:
For example, energy can be saved by decreasing ambient room temperature or by the use of public transportation instead of private cars. Furthermore, the increased use of renewable energy sources contributes to greenhouse gas abatement.
After reading this text completely, please confirm by clicking NEXT

Continued: climate round

After each round the decisions of all players are displayed.
Example with 6 players instead of 18 players for a better overview:
Four players have decided to invest into climate protection. Two of them paid €2 and two of them paid €4.

Pseudonym	**Thebe**	**Triton**	**Portia**	**Sinope**	**Himalia**	**Dione**
Decision	**Yes**	**No**	**No**	**Yes**	**Yes**	**Yes**
Change in the player's account	**-2,00**	**0,00**	**0,00**	**-4,00**	**-2,00**	**-4,00**

In total 12 euros were paid for climate protection and thus credited to the climate account. In the real game all 18 players and their decisions are displayed.
After reading this text completely, please confirm by clicking NEXT

The end of the game

At the end of each game (after exactly 10 rounds) the computer compares the climate account with the threshold amount of €360. This threshold amount has to be reached to avoid dangerous climate change. It is reached if each player pays on average €6 per round (this means €2 per member of the group of 3) for climate protection. In this case

€36 per round would be paid into the climate account. If this amount has been assembled in the climate account, each player will obtain whatever is left over in his account. The money will be paid out in cash anonymously under your pseudonym.
If the threshold amount has **not** been assembled in the climate account dangerous climate change occurs with a probability of **90 %**

(thus in 9 out of 10 cases)

followed by serious economic losses. **This is the probability of losing the money in your account and no group member receives a payment.**
There is a probability of **10% (thus in 1 out of 10 cases**) that you will keep the money in your account. The payment will be paid out in cash anonymously under your pseudonym. The computer decides randomly by "throwing a virtual dice" with this probability.
After reading this text completely, please confirm by clicking NEXT

The experiment will start now
After reading this text completely, please confirm by clicking NEXT

(c) 6 representatives treatment:

Welcome to this experiment in which you can earn money!
At the start of the experiment, 40 euros will be credited to your account as your personal endowment. In the course of the game you or a group member can decide whether money from your endowment will be invested or not. At the end of the game the savings in your account will be paid cash to you. All your decisions are anonymous. To ensure this, the computer assigns a pseudonym to you, visible at the bottom left of the screen. These pseudonyms are names of moons in our solar system (Ananke, Telesto, Despina, Japetus, Kallisto etc.). **For the experiment to be successful experiment you are not allowed to talk to the other participants or make yourself noticeable to them in any way.** You must confirm all information

obtained during the course of the game by clicking NEXT.
After reading this text completely, please confirm by clicking NEXT.

For this experiment, the 18 players are assigned to **six groups of 3 players each**. (see group compilation on the sheet on you desk). For the entire duration of the experiment, you belong to the same group of 3. Your group of 3 is the one in which you see your pseudonym. At the beginning one member of each group is determined randomly, acting as the **representative** of that group. Your representative is playing together with the other 5 representatives a game in which she has at her disposal **the money in total** of her group of 3 and decides **alone** whether and how much she invests thereafter in each round (the accounts of all 3 group members are charged equally). So it depends solely on the decisions of your representative if and how much money you get paid after the game. The rules of the game will be explained in detail on the following pages. After the game the next representative of each group of 3 is **chosen actively** by the 3 group members and the same game is played again by the elected 6 representatives of the groups.
Each player describes in a few sentences how she would represent the interests of her group members. Only her group members can see this information during the election. When seeing the 3 election pledges of her group simultaneously each group member can vote for one of them by clicking the respective button. The player receiving the majority of votes is elected. If no player gets more than one vote, the computer decides randomly.
Each player receives another endowment of € 40.
After the second game, the **active selection** of the representative **is repeated**, and the game is played for a third time again, again with 40 euros endowment per person.
After reading this text completely, please confirm by clicking NEXT

In each of the 3 games the respective representative of the group of 3 receives as an addition to his endowment a **wage of 3 euros**. This wage is charged from the current endowment of the other two group members, € 1.50 each.
Now the game is explained in more detail. The 6 representatives are called players.

Please read the following rules carefully. You could be the next randomly determined representative of your group of 3!
Please realize that your representative handles the **total money** of your group of 3 (that is €120 Euros) and not only his own.
After reading this text completely, please confirm by clicking NEXT

In the course of this experiment you will play **exactly 10 climate rounds**.
In each of these rounds you can invest in an attempt to protect the climate and to avoid dangerous climate change. One of the consequences of dangerous climate change will be serious economic loss, which is simulated in this game. In each round of the game all 6 players will be asked **simultaneously:**

"How much do you want to invest in climate protection?"

(Possible answers: 0, 6 or 12 euros)

Only after each player has made his decision all 6 choices are displayed simultaneously on all 18 laptops. Afterwards all contributions will be credited to an account for climate protection.
Of the money that has eventually been invested in climate protection, we will place an advertisement in your local newspaper. This advert will give general information about simple methods for climate protection - methods that everybody can implement without much effort to protect our climate and to assist in avoiding dangerous climate change. The more money we collect the larger and more conspicuous the advertisement will be. If this promotion is successful, sponsors for international advertising campaigns could be eventually mobilized.
After reading this text completely, please confirm by clicking NEXT

Professor Jochem Marotzke, Director of the Max-Planck-Institute for Meteorology in Hamburg will provide expertise about the state of the climate for the advertisement. He will also give some recommendations on how to reduce CO_2-emissions and on how the climate can be protected:
For example, energy can be saved by decreasing ambient room temperature or by the use of public transportation instead of private cars. Furthermore, the increased use of

renewable energy sources contributes to greenhouse gas abatement.
After reading this text completely, please confirm by clicking NEXT

Continued: climate round

After each round the decisions of all players are displayed.
Example:
Here four players have decided to invest into climate protection. Two of them paid €6 and the other two paid €12.

Pseudonym	**Thebe**	**Triton**	**Portia**	**Sinope**	**Himalia**	**Dione**
Decision	**Yes**	**No**	**No**	**Yes**	**Yes**	**Yes**
Change of the player's account	**-6,00**	**0,00**	**0,00**	**-12,00**	**-6,00**	**-12,00**

In total €36 were paid for climate protection and thus credited to the climate account.
After reading this text completely, please confirm by clicking NEXT

The end of the game

At the end of each game (after exactly 10 rounds) the computer compares the climate account with the threshold amount of €360. This threshold amount has to be reached to avoid dangerous climate change. It is reached if each player pays on average €6 per round (this means €2 per member of the group of 3) for climate protection. In this case €36 per round would be paid into the climate account. If this amount has been assembled in the climate account, each player will obtain whatever is left over in his account. The money will be paid out in cash anonymously under your pseudonym.
If the threshold amount has **not** been assembled in the climate account dangerous climate change occurs with a probability of **90 %**

(thus in 9 out of 10 cases)

followed by serious economic losses. **This is the probability of losing the money in your account and no group member receives a payment.**

There is a probability of **10% (thus in 1 out of 10 cases**) that you will keep the money in your account. The payment will be paid out in cash anonymously under your pseudonym. The computer decides randomly by "throwing a virtual dice" with this probability.

After reading this text completely, please confirm by clicking NEXT

The experiment will start now

After reading this text completely, please confirm by clicking NEXT

Sheet each player received to see the assignment of the 18 players to 6 groups of 3 each:

Group compilation

Group 1	Ananke<br>Telesto<br>Despina
Group 2	Japetus<br>Kallisto<br>Metis
Group 3	Galatea<br>Nereid<br>Elara
Group 4	Vestia<br>Leda<br>Setebos
Group 5	Carpo<br>Pasiphae<br>Umbriel
Group 6	Hyperion<br>Deimos<br>Pallene
